# Enabling Hotspot Detection and Public Health Response to the COVID-19 Pandemic

**DOI:** 10.5888/pcd19.210425

**Published:** 2022-06-30

**Authors:** Randi Foraker, Joshua Landman, Ian Lackey, Matthew D. Haslam, Alison L. Antes, Dennis Goldfarb

**Affiliations:** 1 Division of General Medical Sciences, Department of Medicine, Washington University School of Medicine in St. Louis, St. Louis, Missouri; 2 Institute for Informatics, Washington University School of Medicine in St. Louis, St. Louis, Missouri; 3 Division of Computational and Data Sciences, Washington University in St. Louis, St. Louis, Missouri; 4 Department of Health, City of St. Louis, St. Louis, Missouri; 5 Bioethics Research Center, Division of General Medical Sciences, Department of Medicine, Washington University School of Medicine in St. Louis, St. Louis, Missouri; 6 Department of Cell Biology and Physiology at Washington University School of Medicine in St. Louis, St. Louis, Missouri

## Abstract

**Introduction:**

Public-facing maps of COVID-19 cases, hospital admissions, and deaths are commonly displayed at the state, county, and zip code levels, and low case counts are suppressed to protect confidentiality. Public health authorities are tasked with case identification, contact tracing, and canvasing for educational purposes during a pandemic. Given limited resources, authorities would benefit from the ability to tailor their efforts to a particular neighborhood or congregate living facility.

**Methods:**

We describe the methods of building a real-time visualization of patients with COVID-19–positive tests, which facilitates timely public health response to the pandemic. We developed an interactive street-level visualization that shows new cases developing over time and resolving after 14 days of infection. Our source data included patient demographics (ie, age, race and ethnicity, and sex), street address of residence, respiratory test results, and date of test.

**Results:**

We used colored dots to represent infections. The resulting animation shows where new cases developed in the region and how patterns changed over the course of the pandemic. Users can enlarge specific areas of the map and see street-level detail on residential location of each case and can select from demographic overlays and contour mapping options to see high-level patterns and associations with demographics and chronic disease prevalence as they emerge.

**Conclusions:**

Before the development of this tool, local public health departments in our region did not have a means to map cases of disease to the street level and gain real-time insights into the underlying population where hotspots had developed. For privacy reasons, this tool is password-protected and not available to the public. We expect this tool to prove useful to public health departments as they navigate not only COVID-19 pandemic outcomes but also other public health threats, including chronic diseases and communicable disease outbreaks.

**Summary**

**What is known on this topic?**

Having detailed geographic information on patients affected by the COVID-19 pandemic would allow public health professionals to tailor their efforts and improve future outcomes.

**What is added by this report?**

We built a real-time, interactive, street-level visualization of patients with a COVID-19–positive test to show emerging patterns in relation to disease prevalence and demographic characteristics of patients.

**What are the implications for public health practice?**

We expect this tool to aid public health professionals in mapping disease cases with more granularity and gaining real-time insight into COVID-19 hotspot development.

## Introduction

Public-facing maps of COVID-19 cases, hospital admissions, deaths, and vaccination rates are commonly displayed at the state, county, and zip code levels, and low case counts are suppressed to protect confidentiality (1). Although state laws and public health departmental regulations vary, a standard approach is used to suppress case counts of fewer than 10 (2). Geographically, this approach applies to areas smaller than a county (ie, zip codes and US Census tracts), and case counts are not typically suppressed at the county or state level.

An exception to this rule can be made if data are to be used for quality improvement purposes. Public health departments may prepare reports for internal use that do not suppress case counts. However, such reports cannot be publicly disclosed without approval from a public health authority. Small case counts may be released during a public health emergency, in which, for example, a threat of person-to-person transmission of a communicable disease exists and action must be taken to protect public health.

Public health authorities at the local, regional, and state levels are tasked with case identification, contact tracing, and canvasing for educational purposes during a pandemic. Such authorities have been asked to track vaccination implementation geographically to help ensure equitable distribution of vaccines. Given limited resources, conducting these activities across an entire jurisdiction (ie, a county) or zip code area can be daunting. Rather than being assigned to a high-risk zip code in which to perform public health activities associated with the pandemic, authorities would benefit from the ability to tailor their efforts to a particular neighborhood or congregate living facility.

To address the immediate needs of public health authorities to effectively respond to hotspots of infection in real time, we developed an interactive street-level visualization that shows new cases developing over time and resolving after 14 days of infection. The Health Insurance Portability and Accountability Act (HIPAA) considers residential address to be a direct identifier that must be removed for data to be considered de-identified (3). Thus, access to the identifiable data and visualizations must be restricted to authorized personnel who are proficient data stewards, especially given that such data could be used in harmful ways (4). For privacy reasons, this tool is password-protected and not available to the public. In this article, we describe the methods of building a real-time visualization of patients with COVID-19–positive tests, which facilitates timely public health response to the pandemic.

## Methods

We programmed the disease visualization tool using Data-Driven Documents (D^3^, version 6, d3js.org). We chose D^3^ for its flexibility and ability to create an animated, interactive map. Our source data for the visualization included patient demographics (ie, age, race and ethnicity, and sex), street address of residence, respiratory test results for influenza and SARS-CoV-2, and date of test. Our case data were provided by regional health systems (5). Project source code is available at https://github.com/i2-wustl/visualization-ui. 

Additional data that could easily be linked and integrated by zip code included that from the US Census Bureau’s decennial census or estimates from the American Community Survey (6) to provide sociodemographic and socioeconomic context to the visualization, as well as county-level data on prevalence of chronic disease from the Behavioral Risk Factor Surveillance System (7). We also enabled the use of cancer surveillance and diabetes data, of most interest to stakeholders in our region. Although our goal was to present data at a more granular geographic level, many publicly available sources of data limit our ability to present data at smaller units of geography (ie, US Census tract or block group).

Two-dimensional density plots of case counts were computed using kernel density estimation with empirically chosen values for bandwidth and cell size for each zoom level. The resulting visualization was optimized to balance visual comprehension, aesthetics, and computational performance. The density is presented as cases per square kilometer (cases/km^2^) around the census tract centroid. We preprocessed the residential address data using ArcGIS version 10.8.1 (Esri), yielding longitude and latitude coordinates for each record.

For the publication figures, locations underwent 2 masking steps that are described by Haley et al (8). First, a uniform random perturbation was applied in which we added random numbers between −0.01 and 0.01 to each point’s latitude and longitude, which corresponds to approximately 1.7 km^2^. Next, we performed point aggregation in which each point’s latitude and longitude were rounded to the nearest 0.01 km^2^, corresponding to an approximately 0.85 km^2^–spaced grid. However, the points are not shuffled in the tool when authorized users are logged in.

## Results

**Figure 1:**
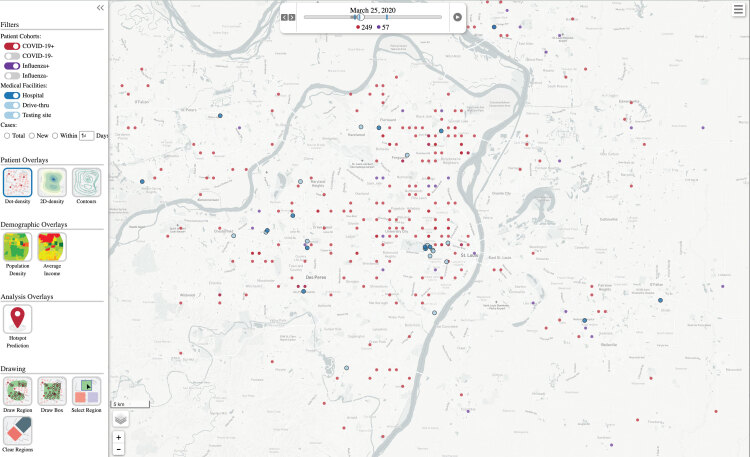
Screenshot of an animation of the disease visualization tool, showing influenza (purple dots) and SARS-CoV-2 (red dots) infections developing and resolving over time. Dark blue dots correspond to hospitals, and light blue dots correspond to testing sites. Case coordinates are shuffled for privacy protection.

The resulting visualization (Figure 1) used colored dots to represent influenza (purple) and SARS-CoV-2 (red) infections. Each dot corresponded to a positive case. Fourteen days after the date of the respiratory test, we considered the case resolved and removed the dot from the visualization. The visualization is activated with a play button and can be paused, rewound, or fast-forwarded to a specific date. The resulting animation shows where new cases developed in the region and how patterns changed over the course of the pandemic. Blue dots corresponded to a hospital (dark blue) or testing site (light blue). The screen shot of the animation shown in Figure 1 has case coordinates shuffled for privacy protection.

The display of case coordinates as well as hospitals and testing sites can be switched on and off (left margin of the visualization) to see only cases of COVID-19 or influenza, for example. Of note, the design of the application is flexible to incorporate other types of health outcomes such as admissions, deaths, and vaccinations. Layers can be added (left margin of the visualization) to show prevalence of chronic disease, sociodemographic characteristics, or socioeconomic characteristics of the zip code or county across the region.

**Figure 2:**
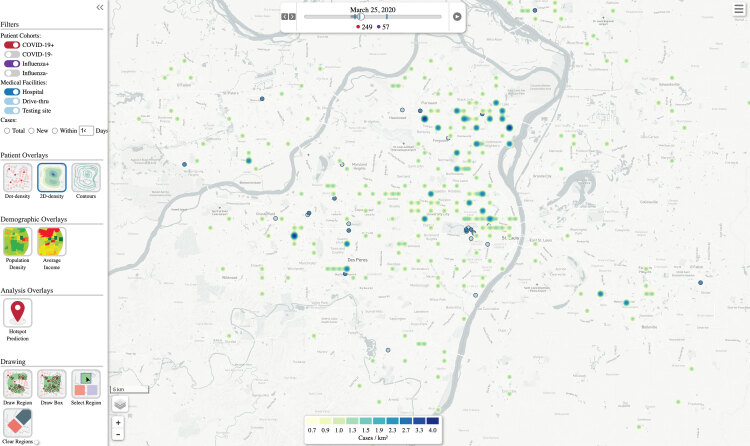
Screenshot of a heatmap animation of the disease visualization tool, showing respiratory virus infection (ie, influenza or SARS-CoV-2) hotspots developing and resolving over time. Case coordinates are shuffled for privacy protection.

Another view of the visualization (Figure 2) uses a heatmap to show how the density of cases per population changes over time. Darker shades correspond to a higher density of cases. As in Figure 1, we resolved cases after 14 days, and the visualization is set in motion with a play button and can be paused, rewound, or fast-forwarded to a specific date. The resulting animation shows where hotspots of COVID-19 developed in the region and how patterns changed over the course of the pandemic. The screen shot of the animation, shown in Figure 2, is based on Figure 1, which had case coordinates shuffled for privacy protection.

We created a secure, password-protected web portal by which approved users can access the application. Users are able to enlarge specific areas of the map and see detail on street-level residential location of each outcome. Tools can be programmed to allow users to select from demographic overlays and contour mapping options to see high-level patterns and associations as they emerge. Additional functionality allows the user to select specific areas on the map to find out more about the demographic distribution of age, race and ethnicity, and sex among outcomes in a particular geographic area. 

## Conclusion

Our efforts are critical given that the COVID-19 pandemic and other public health threats are not respectful of geopolitical boundaries. Publicly available and easily sharable data at the zip code, county, and state levels are not sufficient to enable a precise public health pandemic response. We demonstrated that an open-source solution can be applied to support public health authorities in conducting their case identification and contact tracing activities in the midst of a crisis.

Before the development of this tool, local public health departments did not have a means to map cases of disease to the street level and gain real-time insights into the underlying population where hotspots had developed. The data visualization tool we created addresses this gap and is expected to provide the necessary data-driven insights that will facilitate a timely public health response to the pandemic.

Feasibility assessments should be conducted to evaluate whether the tool meets the needs of the end users and to ensure that sufficient resources are available to act on the disease hotspots that are detected by the tool. Following implementation of the data visualization tool, its utility should be assessed to determine if additional functionality is needed or if the tool can be expanded or in some cases simplified to meet the demands of stakeholders during a pandemic.

A strength of our visualization is that it can be programmed to display other types of public health and COVID-19 outcomes such as hospital admissions, deaths, and vaccinations. We created a lightweight, secure application to address the immediate needs of our region in terms of case identification and contact tracing. We expect this tool to prove useful to public health departments as they navigate not only the COVID-19 pandemic but also other public health threats and communicable disease outbreaks.

Realizing the potential benefits of mapping infections to the street level requires attending to legal issues and ethical responsibilities and mitigating potential risks. Tracking infection at this level of granularity could infringe on individual rights to privacy and confidentiality, which must be balanced by the benefits of this activity for public good (9). As previously stated, HIPAA specifies residential address as a direct identifier (3), and only authorized personnel should have access (4). Data accuracy and validation must be addressed for the tool to realize its intended utility (10). Given that many data sets are incomplete and that data sharing among institutions remains a challenge, this should be monitored going forward (5). Public health practitioners should evaluate for benefits and harms to safeguard trustworthiness, while being mindful that harms could be unevenly distributed among different communities (11).

In conclusion, we aim to share these insights in support of a precision public health approach to an ongoing pandemic. This tool could also be scaled in response to other health outcome tracking needs. Legal and ethical considerations must be aligned with this effort to ensure that data are accessed and used appropriately while protecting privacy and confidentiality.
